# Development of Cell-Specific Aptamers: Recent Advances and Insight into the Selection Procedures

**DOI:** 10.3390/molecules22122070

**Published:** 2017-11-27

**Authors:** Kamal Rahimizadeh, Hadi AlShamaileh, Milena Fratini, Madhuri Chakravarthy, Michelle Stephen, Sarah Shigdar, Rakesh N. Veedu

**Affiliations:** 1 Centre for comparative Genomics, Murdoch University, Perth 6150, Australia; rahimizadeh.kamal@gmail.com (K.R.); h.alshamaileh@murdoch.edu.au (H.A.); milena.fratini@murdoch.edu.au (M.F.); m.chakravarthy@murdoch.edu.au (M.C.); mish.96@live.com.au (M.S.); 2 Perron Institute for Neurological and Translational Science, Perth 6005, Australia; 3 School of Medicine, Deakin University, Waurn Ponds, Geelong 3216, Australia; sarah.shigdar@deakin.edu.au

**Keywords:** aptamers, Cell-SELEX, aptamer enrichment, ssDNA, binding assays

## Abstract

Systematic evolution of ligands by exponential enrichment (SELEX) is an established procedure for developing short single-stranded nucleic acid ligands called aptamers against a target of choice. This approach has also been used for developing aptamers specific to whole cells named Cell-SELEX. Aptamers selected by Cell-SELEX have the potential to act as cell specific therapeutics, cell specific markers or cell specific drug delivery and imaging agents. However, aptamer development is a laborious and time-consuming process which is often challenging due to the requirement of frequent optimization of various steps involved in Cell-SELEX procedures. This review provides an insight into various procedures for selection, aptamer enrichment, regeneration and aptamer-binding analysis, in addition to a very recent update on all aptamers selected by Cell-SELEX procedures.

## 1. Introduction

Aptamers are short single-stranded DNA or RNA oligonucleotides that bind to specific targets through three-dimensional structural conformation and with a collection of other forces such as hydrogen bonding, stacking of aromatic rings and Van der Waals interactions [1,2]. This binding of the aptamers to their targets is typically highly sensitive, with binding affinities in the low nanomolar to picomolar ranges [2]. Aptamer length can vary, but usually ranges between 25 and 90 nucleotide bases, and the smallest known aptamer is 12 bases long [1]. Aptamers are conventionally developed by a process referred to as systematic evolution of ligands by exponential enrichment (SELEX) [3,4,5]. In 2012, our group also reported a one-step selection methodology for rapid aptamer selection [6]. The modifiable nature of the starting oligonucleotide libraries increases the number of aptamer structural variants and increases the possibility of successful binding to the target [5,7]. Additionally, through the introduction of appropriate functional groups, both DNA and RNA aptamers can be made more stable with increased half-life in vitro and in vivo [5,7,8]. The modification and synthesis of aptamers is easily achieved by solid-phase oligonucleotide synthesis [9], and this simplicity and effectiveness of oligonucleotide synthesis is attractive to meet diagnostic/therapeutic challenges towards the treatment of various diseases [10]. Before aptamers became a treatment option, antibodies, proteins and peptides were used for targeted therapy [2]. However, the use of antibodies may involve costly development procedures [10], as they are produced by a complex process that requires the use of animals, cells or other in vivo conditions which often results in batch to batch product variation in different yields, and also require frequent optimization [11]. In contrast, aptamers are chemically synthesized, and therefore, can be produced in consistent yields with no product variation [12]. Furthermore, facile modification of the aptamer endows these aptamers with preferable properties such as fast penetration into cells, lack of immunogenicity, reduced side effects and freedom to introduce multiple chemistries and contrast agents without losing its affinities and specificities to the target [1]. These properties are ideal for aptamers to be used as a tool for targeted therapy, drug delivery and molecular imaging [2,9,13]. Typically, tissue-specific delivery of drug molecules including nucleic acid drugs is very challenging, however, the application of aptamers could be an excellent alternative to circumvent this limitation. Although the use of Cell-surface receptor targeting aptamers could achieve tissue-specific drug delivery to a great extent, development of Cell-specific aptamers may be a better choice. In this review, we highlight the recent advances in the development of Cell-specific aptamers, and provide a comprehensive insight into the selection procedures.

## 2. Cell Specific Aptamer Development by Cell-SELEX

Aptamers selected by conventional SELEX involve the use of a large oligonucleotide library of approximately 10^15^ unique sequences that undergo a series of selection cycles against a specific target of choice [14]. Several rounds of binding, partitioning and amplification are repeated until a pool of high affinity aptamers are enriched [15]. Theoretically, aptamers can be developed against a wide range of targets including peptides, proteins, viruses and whole cells, of which aptamers can bind to intra- or extracellular sites [1,9]. Cell-SELEX follows the similar approach as the traditional SELEX, but uses live cells as the aptamer selection target. A major advantage of Cell-SELEX is the conservation of the native conformation of proteins, as opposed with traditional SELEX that uses mainly the recombinant proteins as targets [1].

## 3. Selection

**Figure 1:**
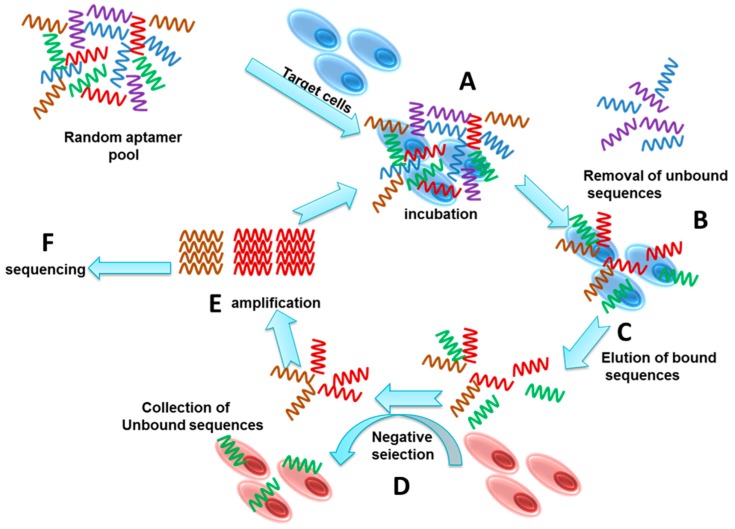
Schematic illustration showing the steps involved in Cell-SELEX. **A**. Incubation of cells with an aptamer library; **B**. Isolating bound sequences from unbound sequences; **C**. Elution of bound sequences; **D**. Negative selection to remove non-specific sequences; **E**. Amplification of target specific sequences; **F**. Sequencing of the selected aptamer pool.

A general SELEX process involves repetitive series of selection cycles starting with the incubation of the target cell with a random sequence single strand oligonucleotides library (Figure 1). Each of the random sequences form unique three-dimensional structures, and the sequences that bind to the target cells are recovered for enrichment and regeneration for subsequent selection cycles, leading to the enrichment of aptamers that bind to the target cells with high affinity and specificity [1]. The enriched library is sequenced to identify the individual bound aptamers.

Consistent cell culture maintenance is critical for Cell-SELEX success, such as consistent confluency across all selection cycles, passage number and growth conditions [16]. Prolonged cell growth or poor culture conditions can influence cell morphology and protein expression and therefore can lead to decreased efficiency of Cell-SELEX [16,17]. Furthermore, to eliminate non-specific binding of oligonucleotides, it is important to block the target cells with tRNA, salmon sperm DNA or Poly (I:C) prior to incubation with the randomized oligonucleotide library [8]. Cell-SELEX can be performed on both adherent and suspension culture. It is important to note that trypsin treatment can strip the cell surface markers and therefore the cells need to be recovered by incubation with the culture media for at least 2 h. Furthermore, if using adherent cells as a cell suspension for aptamer development, the cells should be shaken gently to prevent adhesion of cells [16,17]. Cell-SELEX is performed within the range of 4–37 °C, and increasing selection temperature and extended time increases the possibility of aptamers internalization into cells [17], which is desirable for some aptamer applications.

## 4. Enrichment and Amplification

After each selection cycle, aptamer sequences within the oligonucleotide pool are enriched due to the continuous amplification of high affinity aptamers. However, the amplification of selected aptamers requires optimization to enhance yield without amplifying non-specific sequences due to mispriming. The number of amplification cycles can be optimized by analyzing the PCR products at different amplification cycles by gel electrophoresis. Excessive cycles would result in the amplification of non-specific products, whilst fewer cycles may result in insufficient yields [18]. The number of PCR amplification cycles that results in a band with the highest density without non-specific bands and the lowest amount of smear possible is considered the optimal number of PCR cycles. The optimized amplification cycles number can be used to amplify the aptamer sequences at a larger scale with at least 1 mL of total PCR reaction volume. The PCR product is used to regenerate the library pool for subsequent aptamer selection cycles either by RNA transcription if developing RNA aptamers, or DNA strand separation if developing DNA aptamers [17]. The separation of DNA strands requires a more intricate procedure compared to RNA transcription. In the following section, we focus on four common methodologies used for ssDNA preparation. Although these methods are effective in regenerating ssDNA libraries in SELEX, it is highly recommended to verify the quality of ssDNA with gel electrophoresis.

## 5. Regeneration of Selected Aptamers

### 5.1. Asymmetric PCR

Asymmetric PCR is a simple method for ssDNA preparation in which PCR amplification is carried out with different ratios of primers to preferentially amplify the aptamer sequences without its complementary strand [19,20,21]. In principle, the selected aptamers are amplified into dsDNA during the early PCR amplification cycles. However, the higher amounts of primers that extends the aptamer sequence leads to enrichment of the desired ssDNA [19]. It is important to note that unlike traditional PCR amplification, asymmetric PCR does not exhibit exponential amplification, but rather a linear amplification after depletion of one of the primers. Therefore, it is imperative to optimize the primer ratios and cycle numbers to maximize ssDNA generation. In a case-specific manner, various ratios of primers need to be tested to find the optimum ratio for each SELEX experiment, which is clearly indicated by the highly diverse ratios used in previous Cell-SELEX experiments. Such ratios that displayed efficient ssDNA generation ranged between 15:1 for 20 amplification cycles for Tabarzad et al. [22] and 20:1 for 10–20 amplification cycles for Citartan et al. [19]. Each of these ratios required their own optimization procedure with varying ranges of ratios and different amplification cycle numbers. Indeed, in the case with Tabarzad et al., a small ratio difference between the primers could result in higher yield of dsDNA than ssDNA, while higher ratios (20:1) could lead to higher length sequences due of product-product or product-primer annealing [22]. However, the same ratio (20:1) used by Citartan et al. was found to be optimum for their ssDNA generation. In addition to primer ratio optimization, the number of PCR amplification cycles also contributes to the efficiency of ssDNA generation. Tabarzad et al. [22] stated that it is imperative to determine the highest optimal amplification cycles that guarantees high ssDNA yield and purity without amplification of non-specific products due to opportunistic mispairing. While the amplification parameters established by Citartan et al. and Tabarzad et al. are similar (10–20 amplification cycles), there were successful studies that used much higher amplification cycles for asymmetric PCR up to 30 to 45 cycles with a single primer [19]. Following asymmetric PCR amplification, it is highly recommended to purify the PCR product with gel extraction to eliminate extra primers and any traces of non-specific amplification. Other types of Asymmetric PCR are currently being tested. One such type is the linear-after-the exponential (LATE) PCR in which the length and sequence of the primers can be modified so that the melting temperature of the limiting primer is the same or more than the temperature of the excess primer. This is a promising method for future SELEX experiments [23].

### 5.2. Electrophoresis-Based Separation of DNA Strands

A highly effective method includes the denaturation of the double stranded DNA with urea-containing denaturing polyacrylamide gel electrophoresis (PAGE) [24]. The urea breaks the hydrogen bonds between the two strands, which can be separated on a denaturing PAGE if both strands are of unequal sizes. The enriched aptamer sequences can be identified with UV or blue light transilluminator and subsequently extracted and purified [25,26]. The blue light transilluminator is generally favored over UV for its safety and non-damaging DNA property. Complementary DNA strands usually have equal lengths which would migrate through the denaturing PAGE at equal paces. However, there are various strategies that mitigate this issue. Walder et al. [27] amplified the enriched aptamer sequences with chemically synthesized primers containing ribose residue at the 5′ and 3′ ends. After amplification with the modified primer, the ribose residue can be broken with the ribonuclease enzyme, releasing two separate strands of different lengths and allowing the separation of the aptamer sequence with denaturing PAGE. Separation of complementary strands can also be achieved with linking the aptamer sequence strand with a poly A or T tail with a polyethylene glycol spacer in between, which inhibits DNA polymerization of the complementary to the tail sequence and results in size differences between the strands separable by denaturing PAGE. Alternatively, Keefe et al. [25] added a pH susceptible base on the 3′ end of one of the primers primer, which can be digested with alkaline treatment and creates two different lengths of DNA strands. After separating the aptamer sequence from its complementary strand by denaturing PAGE, the aptamer sequence can be identified and extracted from the gel. While this method is both time-consuming and labor-intensive, it remains a very efficient method for the preparation of ssDNA library for aptamer selection.

### 5.3. Magnetic Beads Separation

**Figure 2:**
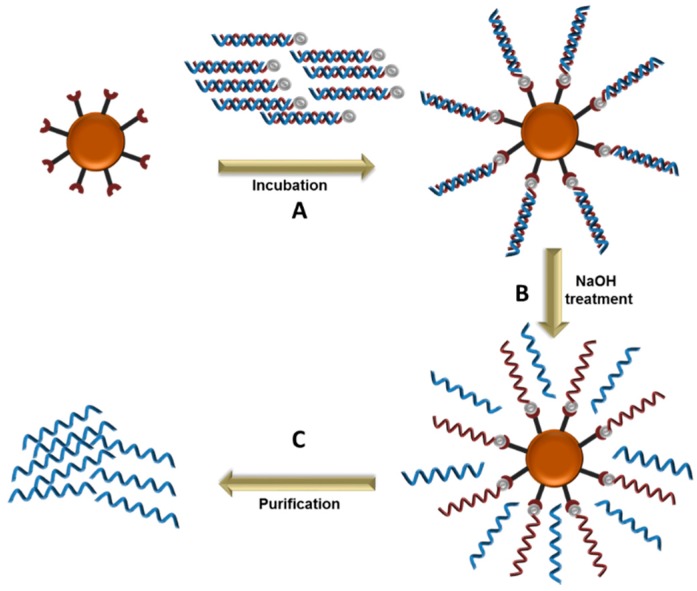
DNA strand separation using magnetic beads. **A**. Immobilization of the double stranded sequences on the magnetic beads; **B**. Denaturation of the double stranded DNA by NaOH treatment; **C**. Purification of the unbound sequences.

Magnetic beads exhibit magnetic properties and congregate when exposed to a magnetic field, which allows for the rapid separation of molecules from a mixture. The amplified aptamer library (dsDNA) are immobilized on streptavidin coated magnetic beads, generally through biotin-streptavidin interaction, allowing for the separation of the DNA strands by increasing the temperature or alkaline treatment (Figure 2). Once immobilized and de-stranded, the immobilized strand is separated from its complementary strand by exposing the magnetic beads to a magnetic field, followed by multiple washings with the recommended buffer. This method has been used in successful Cell-SELEX [28,29]. However, a significant disadvantage is the release the biotin-labeled primer along with the desired ssDNA in the strand separation step, which could overestimate the amount of ssDNA and interfere with aptamer binding to the target molecule. It is therefore recommended to determine the purity of the ssDNA with denaturing gel electrophoresis or mass spectrometry [28].

### 5.4. Lambda Exonuclease Digestion

**Figure 3:**
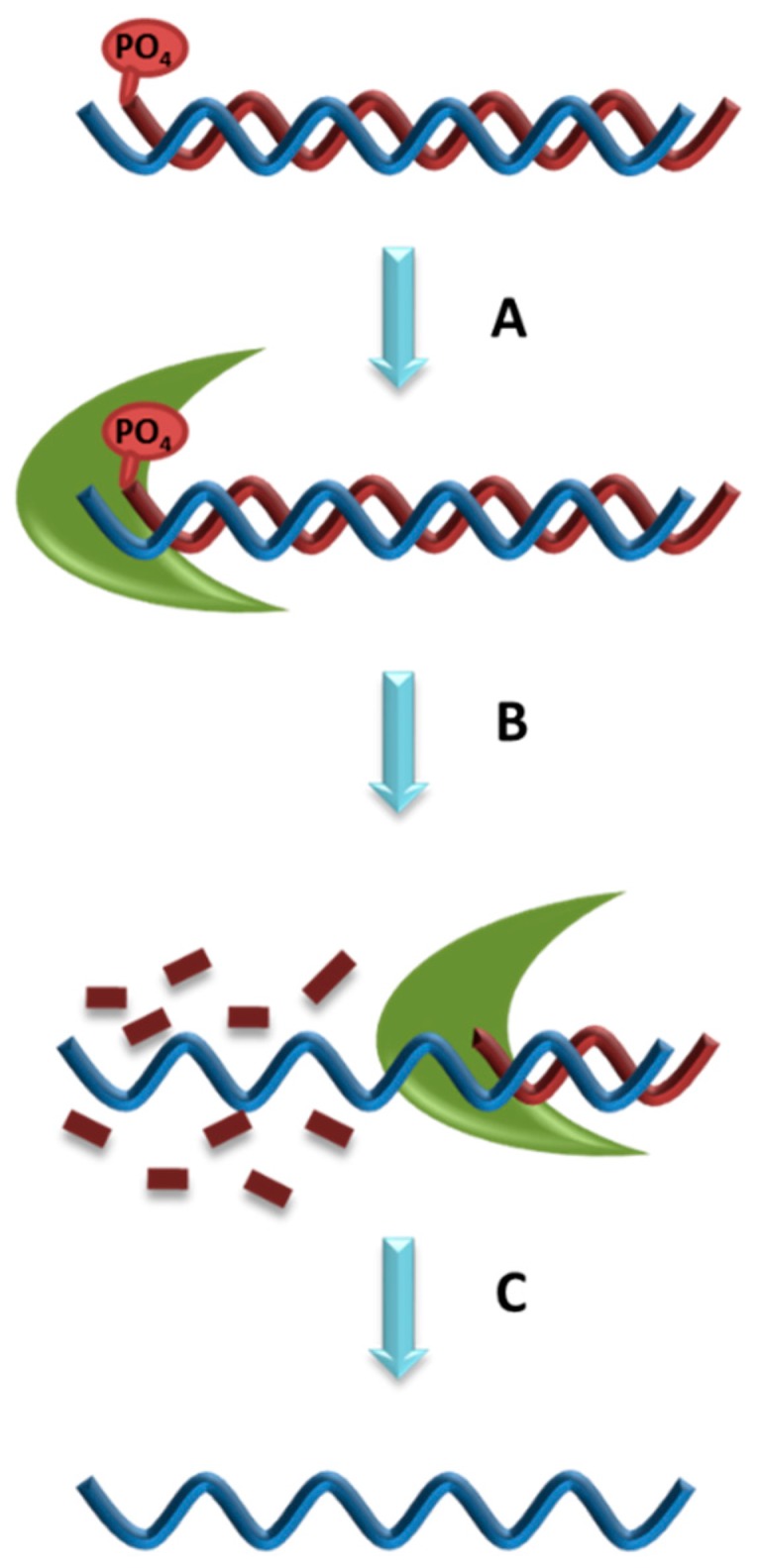
Generation of ssDNA with lambda exonuclease. **A**. Adding enzyme to dsDNA which has a phosphate group; **B**. Incubate enzyme with dsDNA; **C**. Extract and purify the ssDNA.

Lambda exonuclease digests phosphorylated DNA strand from 5′ to 3′ end, leaving the non-phosphorylated ssDNA strand intact [30]. Lambda exonuclease is less active on ssDNA and non-phosphorylated dsDNA and inactive on nicked or gapped DNA, making it an efficient method for ssDNA regeneration for aptamer development [31] (Figure 3). However, extended exposure of ssDNA to lambda exonuclease, even if not phosphorylated, can lead to digestion. Furthermore, low concentration of the lambda exonuclease can be insufficient while high concentration can non-specifically digest DNA [30]. Therefore, it is critical to identify the ideal period of incubation of DNA with lambda exonuclease through a time course analysis [32]. A major disadvantage to lambda exonuclease is the high cost of the enzyme for aptamer development. In addition, after the synthesis has finished, it has to remove not only the phosphate group but also the blocking group which protects the phosphate group. An incomplete digestion of the phosphorylated aptamer leads to accumulation of dsDNA [30].

The ssDNA product from lambda exonuclease digestion requires purification from traces of digested oligonucleotides and the lambda exonuclease enzyme prior to Cell-SELEX progression. Purification can be achieved with phenol chloroform isoamyl alcohol removal followed by ethanol precipitation. However, this process may risk considerable loss of ssDNA yield [31]. Alternatively, the ssDNA can be purified with denaturing PAGE. Regardless of the purification method used, it remains a critical step to run the ssDNA with a denaturing PAGE to confirm the size, yield and digestion efficiency. Purification of ssDNA prevents contamination of digested oligonucleotides and the lambda exonuclease enzyme into the aptamer development process.

## 6. Aptamer Binding Evaluation

Binding assays serve as the first step of confirming the binding efficiency of the newly selected aptamers. It is recommended to perform the binding assays at various stages of the aptamer development process to confirm target-binding enrichment. There are already a number of established binding assays for confirming Cell-specific aptamer binding, such as flow cytometry, fluorescence or confocal microscopy, and enzyme-linked assay. While these methods are different in principle, they share similar steps in binding affinity and specificity determination. Firstly, initial binding affinity tests should be carried out in a similar environment used in the aptamer development process, such as temperature, buffer composition and incubation time. Secondly, it is recommended to block the cells with non-specific oligonucleotide sequences (tRNA, poly I:C, salmon sperm DNA) to block non-specific aptamer binding, which is critical in determining binding specificity. Thirdly, binding affinity can be determined by incubating the target cells with increasing concentrations of the aptamer (generally between 10 and 500 nM), which can be plotted on a non-linear regression curve to determine the dissociation constant (K_d_) of the aptamer. Determination of K_d_ also requires a blank treatment (cells without aptamer treatment, or incubated with the random sequence oligonucleotide library used at the start of Cell-SELEX) to subtract the background noise from the other treatments. If using fluorescence labels, it is recommended to use amber tubes or microplates designed for fluorescence-based assays to minimize photo bleaching. Finally, it is highly recommended to use positive controls, such as aptamers or antibodies that are known to bind to their targets to confirm the efficacy of the binding assay being used.

### 6.1. Flow Cytometry

Flow cytometry is a laser-based method capable of characterizing physical and chemical properties of molecules with high reproducibility and accuracy [33]. Therefore, it has become one of the most common methods in characterizing aptamer binding affinities by passing a stream of single cell suspension through a detection apparatus in single file to detect tagged cells within a population. Indeed, several studies used flow cytometry to demonstrate the binding characteristics of their selected aptamers against a wide variety of cells, including CD133-expressing HEK293T cells [34], acute myeloid leukemia [35], lymphoma [36], *Campylobacter jejuni* [37] and small cell lung cancer [38]. It is necessary to avoid cell clumps with efficient trypsinization and filtration with cell strainers. If trypsinization was a necessary step, the cells need to be incubated with serum-free media for approximately 2 h to recover its cell surface markers.

### 6.2. Enzyme-Linked Assays

Similar to traditional enzyme-linked immunosorbent assays (ELISA) used for specific substance detection, aptamers can be incorporated into enzyme-linked assays to determine their binding affinities, and was first reported in 1996 by Drolet [39] as a highly sensitive and quantitative tool for aptamer binding assay. Furthermore, enzyme-linked assays were also developed specifically for confirming aptamer binding affinities developed by Cell-SELEX, and can be applied to study binding affinities to whole cells, cell lysates, or cell excretions/lysates. Several aptamers developed against microorganisms, such as *Trypanosoma cruzi* excreted secreted antigens (TESA) [40], and human cells, such as U88 glioma cells expressing epidermal growth factor receptor variant III [41], or tumor cells expressing tenascin-C [42] were confirmed through enzyme-linked assays.

### 6.3. Fluorescence and Confocal Microscopy

Although not semi-quantitative, confocal and fluorescence microscopy are among the most popular tools to screen aptamer binding to cells, and can also visually demonstrate the specificity of aptamers towards their targeted cells. While fluorescence and confocal microscopy are similar in principle in the excitation and emission of fluorescence signals, confocal microscopy offers the ability for depth field control, reduction of background and the ability to take serial optical sections. Indeed, aptamer binding affinity and specificity were confirmed with fluorescence and confocal microscopy on human cells, including SK-BR3 expressing human epidermal growth factor receptor-2 (Her-2) [43], HEK cells expressing TrKB [44], leukemia cells [45], HepG2 [46], pancreatic cancer stem cells [47] and HPV-associated cervical cancer cells [48]. In addition to human cells, aptamers developed against microorganisms were also confirmed by fluorescence and confocal microscopy on microorganisms, including *Campylobacter jejuni* [37], trypanosomes [49] and salmonellosis [50]. Both live and stained cells can be used for fluorescence or confocal microscopy, and fluorescence signaling can be achieved by directly adding a fluorescence label to the aptamer, or indirectly by targeting a specific tag on the aptamer with a fluorescently labeled antibody.

### 6.4. Radioactive Scintillation Counting

Scintillation counting offers high sensitivity detection of radiolabeled samples, making it a powerful tool for the detection of aptamer binding reactions to their target cells. For accurate measurements of aptamer binding affinities, separation of aptamer-bound targets from non-binding aptamers is essential and can be achieved by filtration or centrifugation. Aptamer binding assays using scintillation counting was applied to ^32^P-labelled aptamers specific to IVB pili encoded by *Salmonella enterica* serovar Typhi *pil* operon and required filtration of the binding reaction through a nitrocellulose filter, followed by scintillation counting of the nitrocellulose filter [51]. Similarly, aptamers developed for African trypanosomes [49], MCF-7 [52] and differentiated PC12 cells [53] were confirmed by washing away unbound cells and the binding affinity was determined with scintillation counting.

## 7. Aptamers Developed by Cell-SELEX

Traditional SELEX develops aptamers targeting homogeneous molecules, which offers a higher rate of success in selecting high affinity aptamers. However, in biological systems, the use of recombinant proteins as targets away from their native environment risks altering their structural conformation and stability. Native structural conformation is an essential component for aptamer selection intended for therapeutic purposes, and therefore the use of Cell-SELEX is generally favoured as it retains the aptamer selection targets in their natural environment. Although the aptamer targets in Cell-SELEX uses cells in a heterogeneous solution, such as growth media with serum, it remains a highly achievable process as evident by the many Cell-SELEX experiments performed in last few years that resulted in Cell-specific aptamers with high affinity and specificity (Table 1).

## 8. Aptamers Developed Against Cell-Specific Markers

Not all cell-specific aptamers are developed by Cell-SELEX, but can also be developed by traditional SELEX against specific intra- or extra-cellular markers. Aptamers generated against cell markers with traditional SELEX vastly outnumbers aptamers generated by Cell-SELEX. Only a few cell-specific aptamers are presented in this section as more detailed reviews are available [71,72]. Determining the binding affinity aptamers that were developed against a surface cell marker can be achieved with fluorescence/confocal microscopy and flow cytometry, such as the aptamers developed against the Epithelial cell adhesion molecule [73] and CD44 [74]. To determine the binding affinity of aptamers that bind to intracellular targets, it is necessary to lyse the cells to release the aptamers for detection. Furthermore, aptamers released from cell lysis can be immobilized either on plates or magnetic beads to enable enzyme-linked binding assays [39,40,75]. Alternatively, quantitative PCR (qPCR) can be used to detect aptamers in cell lysates by primer-extension [43]. However, it is limited to long sequence aptamers as primers can span between 18 and 22 nt. In addition, surface plasmon resonance (SPR) is an efficient light-based method that can detect aptamer binding via changes in light refraction. Aptamers binding to tenascin-C, a protein found on tumor cells, was detected by passing tenascin-C extracted from tumor cells through a flow cell containing the immobilized aptamers [42].

## 9. Conclusions

The development of cell-specific aptamers carries significant diagnostic and therapeutic potential. Their small size allows for efficient tissue penetration, ease of chemical modification permits longer systemic circulation and high binding affinities allows for rapid molecular detection. However, Cell-SELEX often carries more complications than traditional SELEX that uses pure forms of targets. Unlike the use of purified targets, it is more difficult to direct aptamer binding towards specific parts of the target cell, which may result in the development of non-specific aptamers. The definitive strategy to alleviate the issue of enriching non-specific binding aptamers is the use of a different, but similar, cell line as the negative control selection, one that shares the majority of the target cells’ markers but not the actual aptamer target molecule. In tumor Cell-SELEX, neighboring healthy cells that surround the targeted tumor cells can also serve in negative control selection. Optimized methods for aptamer library regeneration and binding assays play a significant part for the success of Cell-SELEX. Indeed, efficient regeneration and purification of RNA or ssDNA pool is a critical step for aptamer selection as oligonucleotide fragments and dsDNA can nonspecifically bind to cell surfaces and lead to enrichment. Moreover, enrichment of non-specific binding oligonucleotides can potentially give false positive results in non-optimized binding assays. By efficiently regenerating the enriched aptamer pool, non-specific binding can be eliminated from the selection process, and high affinity aptamers are preferentially selected. The DNA strand separation methods described in this review are efficient in regenerating the ssDNA pool for DNA aptamer development, and it is highly recommended to confirm the ssDNA product with gel electrophoresis to confirm the purity and yield. Furthermore, it is critical to use a binding assay method that is compatible with the cell target, such as flow cytometry that scans individual cells in a single-file stream, fluorescence and confocal microscopy for visual aptamer binding confirmation, and scintillation counting for high sensitivity assays. In addition, enzyme-linked assays and qPCR assays allow for the detection of internalized aptamers by analyzing cell lysates or excreted products. Cell-SELEX is a powerful tool, and its successful implementation leads to the development of high affinity aptamers with diagnostic and therapeutic potential, as well as the identification of new cellular markers that advances cell biology research.

## Figures and Tables

**Table 1:** Summary of different Cell-SELEX experiments performed in the last few years.

Aptamer	Method	Target Cell	Kd (nM)	Reference
RNA	Cell-SELEX	HER-2-overexpressing breast cancer	94.6	[43]
DNA	Cell-SELEX	Acute myeloid leukemia cells	5.4 ± 1.6	[35]
RNA	Cell-SELEX	Mouse embryonic stem cells	-	[54]
DNA	Cell-SELEX	Human hepatocarcinoma	19–450	[55]
RNA	Cell-SELEX	AC133-epitope of CD133 expressed on HEK293T cells	33.85–145	[34]
DNA	Cell-SELEX	Over-expressing epidermal growth factor receptor variant III on human glioblastoma	≤100	[56]
DNA	on-chip Cell-SELEX	Different histologically classified ovarian cancer cells	1.3	[57]
DNA	Cell-SELEX	Epidermal growth factor receptor variant III on Glioblastoma	3.37 ± 0.98	[58]
DNA	Cell-SELEX	Metastatic colorectal cell	8.1 ± 0.9	[59]
DNA	Cell-SELEX	Prostate cancer cells	73.59 ± 11.01	[60]
RNA	Cell-SELEX	Annexin A2	10.5 ± 4.6	[61]
DNA	Cell-SELEX By flow cytometry	3T3-L1 adipocytes cells	33.1 ± 2.9	[62]
RNA	Cell-SELEX	PC3-prostate cancer cell	71.57 ± 12.96	[63]
DNA	Cell-SELEX	Human hepatoma cells HepG2	64–349	[64]
DNA	On-chip Cell-SELEX	Colorectal cancer cells	12.3	[65]
DNA	Cell-SELEX	Hepatocellular carcinoma	9.4 ± 2.0	[66]
DNA	Cell internalization SELEX	MCF10CA1h human breast ductal carcinoma		[67]
DNA	Cell-SELEX	Nasopharyngeal carcinoma cell lines	11.93 ± 1.40	[68]
DNA	Cell-SELEX	Renal cell carcinoma (768-O)	9.4 ± 2.0	[69]
DNA	Cell-SELEX	Metastatic colorectal carcinoma LOVO cells	167.3 ± 30.2	[70]
